# Acupuncture Effect and Omics Studies

**DOI:** 10.1155/2014/464206

**Published:** 2014-05-11

**Authors:** Cun-Zhi Liu, Fan-Rong Liang, Jaung-Geng Lin, Sven Schröder, Tiina Rekand, Li Zhu

**Affiliations:** ^1^Acupuncture and Moxibustion Department, Beijing Hospital of Traditional Chinese Medicine Affiliated to Capital Medical University, 23 Meishuguanhou Street, Dongcheng, Beijing 100010, China; ^2^College of Acupuncture and Massage, Chengdu University of Traditional Chinese Medicine, Chengdu, Sichuan 610075, China; ^3^School of Chinese Medicine, China Medical University, Taichung 40402, Taiwan; ^4^HanseMerkur Center for Traditional Chinese Medicine, University Medical Center Hamburg-Eppendorf, Martinistraße 52, 20246 Hamburg, Germany; ^5^Department of Neurology, Haukeland University Hospital, N-5021 Bergen, Norway; ^6^State Key Laboratory of Pathogen and Biosecurity, Beijing Institute of Biotechnology, Beijing 100850, China

The current special issue is the 2013 issue which includes 12 interesting manuscripts.

According to the World Health Organization, acupuncture is useful as adjunct therapy in more than 50 disorders; however, the mechanisms by which acupuncture acts are unclear. In recent years, the omic technologies including genomics, transcriptomics, proteomics, metabolomics/metabonomics, and brain connectomics have been extensively used in acupuncture studies. With these techniques, it seems possible to explore the physiological mechanism of acupuncture in various diseases.

This special issue contains 12 manuscripts, of which 4 systematic reviews are related to the recent advances on human body and diseases under the guidance of holistic view, which will be an utmost important way for developing acupuncture. One manuscript studies the activity of the brachioradialis muscle along the large intestine meridian of hand Yangming by electromyography (sEMG) technology. One manuscript studied the validity of a “Streitberger” needle as a valid approach in a population with experience of acupuncture. Three manuscripts are related to the mechanism of acupuncture. These papers suggested that acupuncture may result from antioxidation, anti-inflammation, and antiapoptosis effects in kinds of diseases. In traditional Chinese medicine, the holistic view not only means the harmonious unity of the whole body, but also includes the unison between man and environment. One manuscript is related to the effect of acupuncture on endometrial immune microenvironment. Two overviews focus on omics application in acupuncture research, which provides an important approach for further exploring the central mechanism of acupuncture.

Omics and systems biology approaches should be particularly good at generating new hypotheses on mechanisms for acupuncture effects, and more research is required to reveal physiological mechanism of acupuncture at the holistic view level.

*Cun-Zhi Liu* *Cun-Zhi Liu* *Fan-Rong Liang* *Fan-Rong Liang* *Jaung-Geng Lin* *Jaung-Geng Lin* *Sven Schröder* *Sven Schröder* *Tiina Rekand* *Tiina Rekand* *Li Zhu* *Li Zhu*

